# Whole genome sequencing identifies associations for nonsyndromic sagittal craniosynostosis with the intergenic region of BMP2 and noncoding RNA gene LINC01428

**DOI:** 10.1038/s41598-024-58343-w

**Published:** 2024-04-12

**Authors:** Anthony M. Musolf, Cristina M. Justice, Zeynep Erdogan-Yildirim, Seppe Goovaerts, Araceli Cuellar, John R. Shaffer, Mary L. Marazita, Peter Claes, Seth M. Weinberg, Jae Li, Craig Senders, Marike Zwienenberg, Emil Simeonov, Radka Kaneva, Tony Roscioli, Lorena Di Pietro, Marta Barba, Wanda Lattanzi, Michael L. Cunningham, Paul A. Romitti, Simeon A. Boyadjiev

**Affiliations:** 1 Statistical Genetics Section, Computational and Statistical Genomics Branch, National Human Genome Research Institute, National Institutes of Health (NIH), Baltimore, MD 21224 USA; 2 Neurobehavioral Clinical Research Section, Social and Behavioral Research Branch, National Human Genome Research Institute, National Institutes of Health (NIH), Bethesda, MD 20892 USA; 3 Center for Craniofacial and Dental Genetics, Department of Oral and Craniofacial Sciences, School of Dental Medicine, University of Pittsburgh, Pittsburgh, PA 15219 USA; 4 Department of Human Genetics, KU Leuven, Leuven, Belgium; 5 Department of Electrical Engineering, ESAT-PSI, KU Leuven, Leuven, Belgium; 6 Medical Imaging Research Center, University Hospitals Leuven, Leuven, Belgium; 7 Department of Pediatrics, University of California Davis, Sacramento, CA 95817 USA; 8 Department of Human Genetics, School of Public Health, University of Pittsburgh, Pittsburgh, PA 15213 USA; 9 Bioinformatics Core, Genome Center, University of California Davis, Davis, CA 95618 USA; 10 Department of Otolaryngology, Head and Neck Surgery, University of California Davis, Sacramento, CA 95817 USA; 11 Department of Neurosurgery, University of California Davis, Sacramento, CA 95817 USA; 12 Pediatric Clinic, Alexandrovska University Hospital, Medical University of Sofia, 1431 Sofia, Bulgaria; 13 Molecular Medicine Center, Department of Medical Chemistry and Biochemistry, Medical Faculty, Medical University of Sofia, 1431 Sofia, Bulgaria; 14 Neuroscience Research Australia, University of New South Wales, Sydney, Australia; 15 Department of Life Sciences and Public Health, Università Cattolica del Sacro Cuore, 00168 Rome, Italy; 16 Fondazione Policlinico Universitario A. Gemelli, IRCCS, 00168 Rome, Italy; 17 Seattle Children’s Craniofacial Center, Center of Developmental Biology and Regenerative Medicine and Division of Craniofacial Medicine, Department of Pediatrics, University of Washington, Seattle, WA 98105 USA; 18 Department of Epidemiology, College of Public Health, The University of Iowa, Iowa City, IA 52242 USA

**Keywords:** Whole genome sequencing, Craniosynostosis, Sagittal suture, Transmission disequilibrium test, Trio study, Sequencing, Genetics research, Paediatric research

## Abstract

Craniosynostosis (CS) is a major birth defect resulting from premature fusion of cranial sutures. Nonsyndromic CS occurs more frequently than syndromic CS, with sagittal nonsyndromic craniosynostosis (sNCS) presenting as the most common CS phenotype. Previous genome-wide association and targeted sequencing analyses of sNCS have identified multiple associated loci, with the strongest association on chromosome 20. Herein, we report the first whole-genome sequencing study of sNCS using 63 proband-parent trios. Sequencing data for these trios were analyzed using the transmission disequilibrium test (TDT) and rare variant TDT (rvTDT) to identify high-risk rare gene variants. Sequencing data were also examined for copy number variants (CNVs) and de novo variants. TDT analysis identified a highly significant locus at 20p12.3, localized to the intergenic region between *BMP2* and the noncoding RNA gene *LINC01428*. Three variants (rs6054763, rs6054764, rs932517) were identified as potential causal variants due to their probability of being transcription factor binding sites, deleterious combined annotation dependent depletion scores, and high minor allele enrichment in probands. Morphometric analysis of cranial vault shape in an unaffected cohort validated the effect of these three single nucleotide variants (SNVs) on dolichocephaly. No genome-wide significant rare variants, de novo loci, or CNVs were identified. Future efforts to identify risk variants for sNCS should include sequencing of larger and more diverse population samples and increased omics analyses, such as RNA-seq and ATAC-seq.

## Introduction

Craniosynostosis (CS), a major structural birth defect, is the premature fusion of one or more cranial sutures affecting 1 in 2500 live births^1^. Approximately 80% of children diagnosed with CS present as an isolated phenotype (i.e. nonsyndromic CS [NCS])^2^, with the suture fusion being the only defect. The remainder of children with CS present with syndromic CS that includes additional major birth defects and/or developmental delays. The most common form of NCS involves the sagittal suture and comprises 40–58% of all NCS cases^3^ with a prevalence of 1.9–2.3 per 10,000 live births^4^. The fusion of the sagittal sutures can result in a long and narrow skull shape, known as scaphocephaly.

CS is considered to have a multifactorial etiopathogenesis. Variants in at least 10 genes (*FGFR1*, *FGFR2*, *FGFR3*, *TWIST1*, *RAB23*, *EFNB1*, *TCF12*, *MSX2*, *POR, ERF*) are associated with Mendelian forms of syndromic CS^5–7^. Syndromic CS has also been associated with a number of chromosomal aberrations^8^. Conversely, most children with NCS present as sporadic cases, although an estimated 6–8% of families have multiple affected individuals^3^. Segregation analyses of multiplex NCS families support an autosomal dominant inheritance with 38% penetrance for sagittal NCS (sNCS)^4^ and 60% penetrance for coronal NCS (cNCS)^9^.

The etiopathogenesis of NCS represents a large gap in our current knowledge, but findings from high-throughput genomic studies have begun to narrow this gap. From the two genome-wide association studies (GWASs) to date, novel and replicated associations to a variant near *BMP2* (rs1884302) and in *BBS9* (rs10262453) were detected for sNCS^10^ and a novel and replicated association to a variant in *BMP7* (rs6127972) was identified for metopic (mNCS)^11^. An experimental study also demonstrated that rs1884302 in *BMP2* acts as an enhancer in a zebrafish model^12^. In addition, three whole-exome sequencing (WES) studies have identified *SMAD6* loss-of-function (LOF) mutations in 7% of midline NCS probands^13,14^, mutations in *ERF* in 5 of 12 NCS multiplex families^15^, and mutations in *TCF12* in 10% of unilateral cNCS and 32% of bicoronal NCS probands^16^.

Whole-genome sequencing (WGS) data provide even more opportunity to identify potential causal variants in CS. Unlike SNV arrays used for GWAS studies, which only contain portions of the genome, and WES, which focuses purely on the exome, WGS targets the entire genome. This allows coverage of rare variants in potential transcription factor binding sites, enhancers, silencers, splicing sites, expression quantitative trait loci (eQTLs, i.e. variants influencing gene expression), and other noncoding regulatory elements that might affect disease risk yet would not be identified in either GWAS or WES studies. WGS has been effectively used to identify new genetic loci across multiple diseases, including neurodegenerative diseases (e.g. Alzheimer’s disease^17^ and Lewy body dementia^18^) and behavioral diseases (e.g. schizophrenia^19^ and autism^20^). WGS has also been used to identify deletions in patients with CS^21^.

Herein, we report on the first study to use WGS to identify genetic loci associated with sNCS. Our dataset is trio-based, specifically looking at affected proband-unaffected parent trios. Proband-parent trio data were analyzed by the transmission equilibrium test (TDT), which tests for genetic association in the presence of genetic linkage, and is robust to population stratification^22^. Multiple extensions of TDT exist, including those that utilize quantitative traits^23^ or rare variants (RVs)^24^. In this study, we use TDT and rare variant TDT (rvTDT) to analyze WGS data from 63 proband-parent trios with sNCS. Our analyses identified a highly associated intergenic region on chromosome 20 between *BMP2* and the noncoding RNA gene *LINC01428*.

## Materials and methods

### Study population

A total of 100 probands diagnosed with sNCS and their unaffected parents were selected for this study from the cohort of ~ 1500 families with NCS that were recruited through the collaborative efforts of investigators in the International Craniosynostosis Consortium (https://health.ucdavis.edu/pediatrics/research/labs/boyd-genetics-lab/craniostudy). This sample included 23 trios with variants significantly associated with sNCS in our previous GWAS. The study was approved by the Institutional Review Board of the University of California Davis and the corresponding entities at each collaborating site. The study protocol was conducted in accordance with the guidelines required by these approvals, including obtaining signed informed consents from all study participants prior to review of medical documentation, clinical examinations, and specimen collection. Evaluation of probands and their parents was conducted via clinical examination by a clinical geneticist; no proband was found to have additional major birth defects or developmental delays, and no parent was identified to have a major birth defect. DNA was extracted from blood, saliva, or mouthwash according to the manufacturer's protocol using the Gentra Puregene Blood Kit (QIAGEN). All proband specimens were tested and were negative for variants in the hot spots of *FGFR1*, *FGFR2*, *FGFR3* and the entire *TWIST1* gene, as described elsewhere^25^. DNA from a total of 63 proband-parent trios successfully underwent WGS as described below; 7 of these trios included additional related individuals with sNCS.

### Sequencing and quality control

Library construction and sequencing was performed by HudsonAlpha Discovery, a division of Discovery Life Sciences. Specifically, 3–5 µg of genomic DNA was sheared using a Megaruptor 3 (Diagenode) and purified using Ampure XP beads. Sheared DNA was size selected using the PippinHT instrument (Sage Science) with a target range of 16–20 kb fragments. Next, 1 µg of fragmented, purified, and size-selected DNA in a volume of 47 µl was used in the SQK-LSK109 library preparation protocol per manufacturer’s instructions (Oxford Nanopore Technologies). DNA was end-repaired using the NEBNext FFPE DNA Repair Mix and NEBNext Ultra II End Repair/dA-tailing modules, followed by purification with AMPure XP beads (1:1 vol ratio) and elution to a final volume of 60 µl. Adapters were ligated, and the final library resuspended in Long Fragment Buffer (Oxford Nanopore Technologies). The resulting final library yield was 1.2–2.2 µg per specimen. Libraries were loaded onto PromethION Flowcells (R9.4.1) with 20 femtomolar (fM) loading. After 24 h, all specimens were nuclease washed and reloaded with 20 fM of library. Total sequencing run time was 72 h.

GVCF (a g compressed VCF file) files were created for 63 sNCS families (196 individuals) using Genome Analysis Tool Kit (GATK) applying their best practices^26,27^. Additional quality control was performed using PLINK^28^. Monomorphic variants (n = 10,341), as well as those with a call rate below 99% (n = 15,678), were removed. Identity-by-descent (IBD) calculations to ensure all familial relationships resulted in removal of one individual from a four-person family; the proband-parent trio remained intact. Mendelian inconsistencies that were localized to a single family were removed from that family only; any Mendelian error that was present in multiple families was removed from the entire dataset. Mendelian inconsistencies were evaluated separately as potential de novo variants. Copy number variant (CNV) calling was performed using CANOES^29^.

### TDT analysis

Sequencing data for the 63 families were analyzed using TDT in PLINK^28^, with testing being performed between each variant and sNCS. Seven of the families had sib-pairs; a proband-parent trio was extracted from those families by selecting the three individuals with the highest genotype call rates that formed the complete trio. In total, 189 individuals (63 trios) were analyzed by TDT. Two stratified analyses were also performed. The first stratified analysis focused on families with only one affected child; thus, the 21 individuals (7 trios) that were extracted from the multiplex families were removed, leaving 168 individuals (56 trios). The second stratified analysis was to examine results in families not included in our original published GWAS to serve as an internal replication for our WGS analysis; thus, the 69 individuals (23 trios) that were used in our original GWAS were removed, leaving 40 trios with 120 individuals.

### Rare variant TDT analysis

To leverage RVs in our WGS dataset, gene-based versions of the TDT (ran through rvTDT^24^) were used to capture RVs that might be underpowered in our traditional TDT analysis. The rvTDT uses collapsing methods (including combined multivariate collapsing [CMC] and variable threshold [VT] methods) to create a gene-based marker that corresponds to a gene or intergenic region for TDT analysis as a single marker. RVs were defined as those in our dataset with a minor allele frequency (MAF) ≤ 0.02 in the non-Finnish European gnomAD population.

### Variant annotation

Variants were annotated using wANNOVAR^30^. Of interest were potential RVs in coding regions, with rare being defined as a MAF < 1% in gnomAD non-Finnish European database, as these variants would not be analyzed under the traditional TDT analysis. Noncoding variants were annotated using RegulomeDB^31^ with the goal of determining potential transcription factor binding sites. CADD scores^32–34^ and ClinVar data^35^ were used in annotation.

### Effect of sNCS risk variants on cranial vault shape

The effects of variants nominated through WGS on normal-range human cranial vault shape were interrogated. These data were generated by leveraging imaging (whole head magnetic resonance [MR] scans) and SNV array (Affymetrix NIDA Smokescreen™) on 6772 individuals available through the Adolescent Brain Cognitive Development (ABCD) study cohort (Data release 3.0)^36^. The working hypothesis was that variants impacting sNCS risk will also lead to a tendency toward a dolichocephalic vault shape in the general population. The hypothesis was tested by examining the effects of sNCS risk variants separately and as haplotypes (when possible) on measures of 3D vault shape based on a recently completed GWAS (Goovaerts et al.^37^). This GWAS contains a detailed description of the methods used to derive the 3D vault phenotypes from MR scans and the subsequent investigation of SNV effects on vault shape.

## Results

### Analytical sample

The final cleaned dataset contained 33,470,718 SNVs and indels in 63 sNCS families, comprised 196 individuals (63 probands, 7 affected sibs, and 126 unaffected parents). All individuals were of non-Finnish European ancestry collected from the United States, Australia, Bulgaria, and Italy. Our initial TDT analysis removed the 7 affected sibs leaving 63 probands (109 males; 57% male) and 126 unaffected parents for analysis.

### TDT analysis

**Figure 1:**
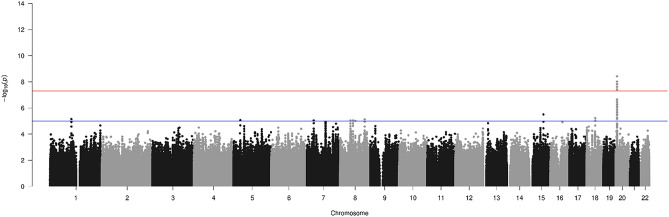
Genome-wide Manhattan plot of full transmission disequilibrium test analysis for 63 sagittal nonsyndromic craniosynostosis trios. The lines at 7.3 and 5 represent the respective thresholds for genome-wide significant and genome-wide suggestive associations.

Twenty-four genome-wide significant (GWS) variants (<5e − 8) were identified from our TDT analysis (Fig. 1, Table 1). All variants were located on chromosome 20p12.3 in the intergenic region between *BMP2* and the noncoding RNA gene *LINC01428*. Each variant identified had an OR > 4.8, and all variants were common (MAF ≥ 0.390). The lead variants were rs6054748 and rs1884302, with identical p-values and odds ratios (ORs) of 3.80e − 9 and 5.5, respectively. Eight statistically suggestive variants (p-value < 1e − 5) were identified on chromosomes 1, 5, 7, 8, 15, and 18 (two independent suggestive variants each on chromosomes 7 and 8).

**Table 1:** Genome-wide significant variants from transmission disequilibrium test analysis.

Chr	SNP	Pos	Min allele	Maj allele	OR	P	Conseq	Gene	MAF	TFBS	CADD
20	rs6054748	7123941	A	G	5.5	3.80E − 09	intergenic	BMP2;LINC01428	0.476	0.18	1.57
20	rs1884302	7125642	C	T	5.5	3.80E − 09	intergenic	BMP2;LINC01428	0.476	0.00	2.13
20	rs6054761	7135601	G	T	5.4	9.63E − 09	intergenic	BMP2;LINC01428	0.396	0.13	0.37
20	rs6054763	7137160	C	G	5.4	9.63E − 09	intergenic	BMP2;LINC01428	0.396	0.61	6.75
20	rs932517	7137588	G	A	5.4	9.63E − 09	intergenic	BMP2;LINC01428	0.396	0.59	4.07
20	rs6117638	7126418	C	T	5.1	9.75E − 09	intergenic	BMP2;LINC01428	0.480	0.17	1.84
20	rs1124471	7130193	A	C	5.1	9.75E − 09	intergenic	BMP2;LINC01428	0.480	0.18	0.42
20	rs6054756	7132317	A	G	5.1	9.75E − 09	intergenic	BMP2;LINC01428	0.480	0.45	0.96
20	rs6054759	7133639	C	T	5.1	9.75E − 09	intergenic	BMP2;LINC01428	0.480	0.33	0.19
20	rs6054764	7137945	C	T	5.1	9.75E − 09	intergenic	BMP2;LINC01428	0.480	0.61	9.99
20	rs2876076	7142331	C	G	5.1	9.75E − 09	intergenic	BMP2;LINC01428	0.480	0.13	1.53
20	rs985051	7134640	A	G	5.3	1.53E − 08	intergenic	BMP2;LINC01428	0.390	0.18	1.96
20	20:7134759	7134759	A	AC	5.3	1.53E − 08	intergenic	BMP2;LINC01428	0.390	n/a	
20	rs1159530	7123062	C	G	5.0	1.54E − 08	intergenic	BMP2;LINC01428	0.492	0.13	0.34
20	rs2326896	7138844	C	A	5.0	1.54E − 08	intergenic	BMP2;LINC01428	0.476	0.23	0.97
20	rs2876077	7142513	C	T	5.0	1.54E − 08	intergenic	BMP2;LINC01428	0.484	0.23	1.07
20	rs1401362781	7144987	C	CA	5.0	1.54E − 08	intergenic	BMP2;LINC01428	0.396	0.55	
20	20:7145408	7145408	A	AAAAT	5.2	2.43E − 08	intergenic	BMP2;LINC01428	0.395	n/a	n/a
20	rs6054747	7123915	T	C	4.9	2.44E − 08	intergenic	BMP2;LINC01428	0.400	0.18	1.11
20	rs6140184	7124556	G	C	4.9	2.44E − 08	intergenic	BMP2;LINC01428	0.400	0.18	2.96
20	20:7125391	7125391	G	GAAAAC	4.9	2.44E − 08	intergenic	BMP2;LINC01428	0.400	n/a	n/a
20	rs1884303	7125706	G	A	4.9	2.44E − 08	intergenic	BMP2;LINC01428	0.400	0.18	3.12
20	rs1159531	7122948	A	G	4.6	3.78E − 08	intergenic	BMP2;LINC01428	0.408	0.24	3.40
20	20:7138834	7138834	A	AT	4.8	3.84E − 08	intergenic	BMP2;LINC01428	0.394	n/a	n/a

Headers represent – CHR = chromosome, SNP = SNP rsID, Pos = position in base pair, Min allele = minor allele, Maj allele = major allele, OR = odds ratio, P = p-value, Conseq = variant consequence, MAF = minor allele frequency, TFBS = probability of TF binding site, CADD = CADD score.

**Figure 2:**
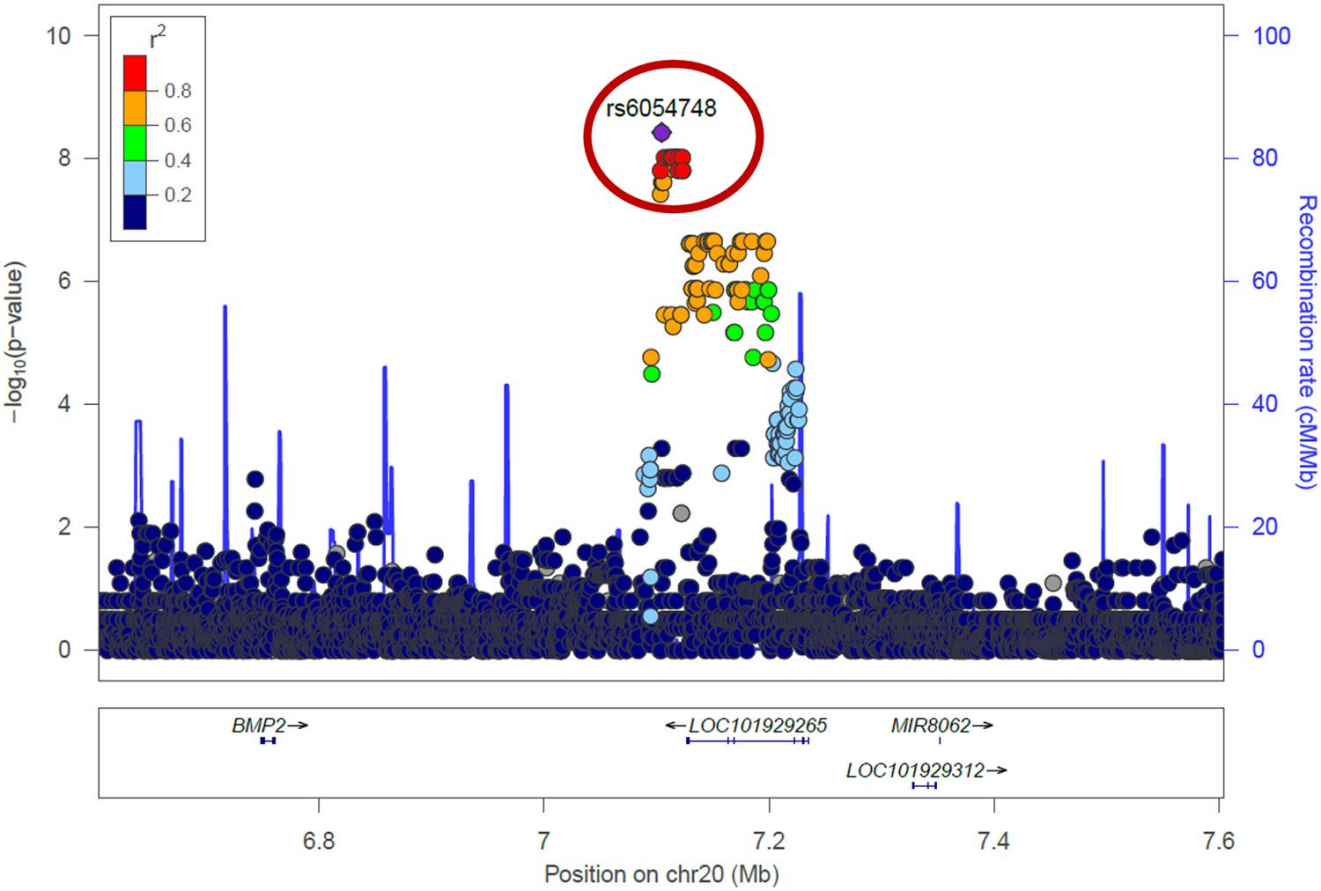
Zoomed in plot of the genome-wide significant region at 20p12.3. Manhattan plot zoomed in on the chromosome 20 linkage signal. rs1884302 (not shown) is in complete linkage disequilibrium with rs6054748 (shown) within the haplotype containing these variants (red circle). LOC101929265 is an alias for LINC01428.

The analytical sample used to identify associations for rs6054748 and rs1884302 included some of the trios from our previous GWAS^10^. These trios were selected for sequencing based on carrying this variant to allow us to examine a much more granular view of these loci and the potential for identifying the true causal variant. A closer look at the GWS variants on chromosome 20 shows a small, linked haplotype containing these variants localized to a 30,000 bp region flanked by rs1159531 (7,122,948 bp) to rs1401362781 (7,144,987 bp) (Fig. 2).

**Figure 3:**
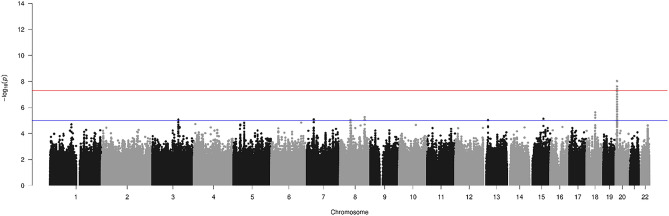
Genome-Wide Manhattan Plot of Transmission Disequilibrium Test Analysis with Multiplex Families Removed (56 remaining Sagittal Nonsyndromic Craniosynostosis Trios). The lines at 7.3 and 5 represent the respective thresholds for genome-wide significant and genome-wide suggestive associations.

To ensure that the GWS variants identified on chromosome 20 were not being driven by the seven multiplex families from which we had extracted a proband-parent trio, we reran association tests excluding these trios. Even with the slight reduction in statistical power expected due to decreased sample size, 10 variants remained GWS (Fig. 3) These 10 variants were a subset of the original 24 GWS variants from the 63 trios. No other GWS variants were observed. The two variants with the highest significance remained rs1884302 and rs6054748 (p-values = 9.36e − 9; ORs = 5.7) (Table 2).

**Table 2:** Genome-wide significant variants from transmission disequilibrium test analysis with multiplex families removed.

Chr	SNP	Pos	Min allele	Maj allele	OR	P	Conseq	Gene
20	rs6054748	7.12E + 06	A	G	5.7	9.36E − 09	Intergenic	BMP2;LINC01428
20	rs1884302	7.13E + 06	C	T	5.7	9.36E − 09	Intergenic	BMP2;LINC01428
20	rs6117638	7.13E + 06	C	T	5.2	2.43E − 08	Intergenic	BMP2;LINC01428
20	rs1124471	7.13E + 06	A	C	5.2	2.43E − 08	Intergenic	BMP2;LINC01428
20	rs6054756	7.13E + 06	A	G	5.2	2.43E − 08	Intergenic	BMP2;LINC01428
20	rs6054759	7.13E + 06	C	T	5.2	2.43E − 08	Intergenic	BMP2;LINC01428
20	rs6054764	7.14E + 06	C	T	5.2	2.43E − 08	Intergenic	BMP2;LINC01428
20	rs2876076	7.14E + 06	C	G	5.2	2.43E − 08	Intergenic	BMP2;LINC01428
20	rs2326896	7.14E + 06	C	A	5.1	3.85E − 08	Intergenic	BMP2;LINC01428
20	rs2876077	7.14E + 06	C	T	5.1	3.85E − 08	Intergenic	BMP2;LINC01428

Headers represent – CHR = chromosome, SNP = SNP rsID, Pos = position in base pair, Min allele = minor allele, Maj allele = major allele, OR = odds ratio, P = p-value, Conseq = variant consequence.

We also wanted to examine how much of the association signal at this locus was driven by the 40 trios included in our WGS that were not included in our prior GWAS. Among this reduced sample set, no variants were identified as GWS, although three variants in complete linkage disequilibrium (LD) rs6054761, rs6054763, and rs932517 showed the strongest signal with identical p-values and ORs (p = 1.54e − 5, OR = 5.5). Only two variants on chromosome 17 near *KCNJ2* showed lower p-values (Table 3).

**Table 3:** Top five significant variants from transmission disequilibrium test analysis with trios included in our previous genome-wide association study removed.

Chr	SNP	Pos	Min allele	Maj allele	OR	P	Type	Gene
17	rs1877742	70197503	T	C	6.4	9.05E − 06	intergenic	KNCJ5
17	n/a	70202707	G	GAGA	6.2	1.47E − 05	intergenic	KNCJ5
20	rs6054761	7135601	G	T	5.5	1.54E − 05	intergenic	BMP2;LINC01428
20	rs6054763	7137160	C	G	5.5	1.54E − 05	intergenic	BMP2;LINC01428
20	rs932517	7137588	G	A	5.5	1.54E − 05	intergenic	BMP2;LINC01428

Headers represent – CHR = chromosome, SNP = SNP rsID, Pos = position in base pair, Min allele = minor allele, Maj allele = major allele, OR = odds ratio, P = p-value.

### rvTDT

The rvTDT analysis on our 63 trios did not yield any GWS results at a GWS threshold of 1e-5. The region with the highest significance was observed between *LL22NC01-81G9.3* and *TEX33* on chromosome 22 (p-value = 3.5e − 5), and the gene with the highest significance was *HOXB13* on chromosome 17 (p-value = 2e − 4) (Supplemental Table 1).

Although our GWS locus on chromosome 20 was driven by common variants, we explored whether these variants might be tagging rare causal variants; however, the intergenic region between *BMP2* and *LINC01428* was not significant (p-value = 0.99). As such, it is likely that the association signal at this locus is driven solely by common variation.

### CNV and de novo analysis

No GWS CNVs (<5 e − 8) were identified, and no significant de novo variants were found.

### Causal variant identification

No GWS variants identified in our TDT analysis were in coding regions, making identification of potential causal variants more challenging. One approach for determining causality for noncoding variants is to cross-reference association results with functional annotations from reference datasets, including variants known to affect RNA expression (eQTLs), splicing (sQTLs), or protein expression (pQTLs). No variant in our identified haplotype was found to be a known QTL of any kind. Another approach is determining if a variant is located in a likely transcription factor binding site (TFBS). Three variants (rs6054763, rs6054764, rs932517) that are clustered close together with less than 800 bp among them had a probability of 55–61% of being a TFBS. Chromatin immunoprecipitation (ChIP) data from RegulomeDB revealed potential binding for multiple TFs for each of these variants (Table 4). *TBX21* and *MEF2B* are shared between rs6054764 and rs932517 and *FOS* is shared between rs6054763 and rs6054764.

**Table 4:** Potential binding transcription factors based on ChIP results for best candidate variants.

SNP	Transcription factors
rs6054763	MTA2, ZNF217, FOS, JUND
rs932517	TBX21, MEF2B
rs6054764	CTCF, DPF2, TBX21, RELB, BHLHE40, RAD51, MEF2B, FOS, TBP, IKZF2, IKZF1, MNT, EP300

Header represents—SNP = SNP rsID.

CADD scores can be used to predict the potential deleteriousness of non-coding variants, as they incorporate additional annotations besides protein prediction, such as conservation, DNase hypersensitivity, and splicing sites into their scores^32–34^ (Table 1). The three variants with the highest CADD scores were the same three variants with the highest TFBS binding scores, rs6054763 (CADD score 6.75), rs6054764 (CADD score 9.99), and rs932517 (CADD score 4.07). Additionally, the minor allele frequency of each variant was highly enriched in our sNCS cohort compared to the general population, with rs6054764 enriched by 16% and rs6054763 and rs932517 enriched by 11% compared to gnomAD non-Finnish Europeans (Table 5). Further, as shown by the results of our analysis removing the trios used in our original GWAS, these SNVs were the most significant along the haplotype in the 40 new trios. Taking the TFBS, CADD, and allele enrichment evidence into account, these three closely clustered variants within this 800 bp region seem to be the most promising candidates for causality. Linkage disequilibrium (LD analysis brings the total haplotype to about 1100 bp; the entire set of GWS variants lie on a haplotype of 14,893 bp.

**Table 5:** Differences in minor allele frequency between dataset cohort and general population in candidate variants.

SNP	Dataset MAF	gnomAD NFE MAF	Difference
rs6054763	0.396	0.29	0.106
rs932517	0.396	0.29	0.106
rs6054764	0.480	0.32	0.16

Headers represent—SNP = SNP rsID, Dataset MAF = Dataset minor allele frequency, gnomAD NFE MAF = Genome Aggregation Database non-Finnish Europeans minor allele frequency.

### Haplotypes of sNCS risk variants impact cranial vault shape in the general population

With variation near *BMP2* being implicated in both sNCS and normal-range variation in cranial vault shape, we aimed to investigate how the locus comprising sNCS risk variants rs6054763, rs932517, and rs6054764 affected cranial vault shape in the general population based on a recent GWAS (Goovaerts et al.^37^) in a multi-ancestry cohort of 6772 adolescents. Each variant was GWS and presented dolichocephalic tendency for the minor allele with very similar overall shape effects across the variants due to high LD (Supplemental Fig. 1).

To further elucidate how variation at this locus relates to cranial vault shape variation in the general population, we investigated how haplotypes of sNCS risk variants rs6054763, rs932517, and rs6054764 affect cranial vault shape. Among ABCD participants with recent European ancestry (N = 5746), rs6054763 (G/C), rs932517 (A/G), and rs6054764 (T/C) constitute three distinct haplotypes: CGC, GAT, and GAC with haplotype frequencies of 28%, 68%, and 4% respectively, closely matching those in the European populations of the 1000 Genomes Phase 3 dataset (28%, 67%, and 5%). ABCD participants were stratified according to diplotype (i.e. the haplotype pair on homologous chromosomes; six possible combinations) and for each group we extracted 3D cranial vault shape from T1-weighted MR images as previously described (Goovaerts et al.^37^). After adjustment for covariates (age, sex, height, weight, cranial size, 10 genetic ancestry principal components, and the model of MR scanner used), the average cranial vault shape of each group was compared with that of the homozygous GAT carriers as the reference group because this haplotype was devoid of any sNCS risk alleles. Furthermore, cranial index was measured from adjusted 3D cranial vault shape for each individual as the ratio of maximum cranial width and maximum cranial length. For the CGC and GAC haplotypes, association with cranial index was assessed by a two-sample t-test (two-tailed), testing the null hypothesis that carriers of at least one index haplotype share the same average cranial index as homozygous GAT carriers.

**Figure 4:**
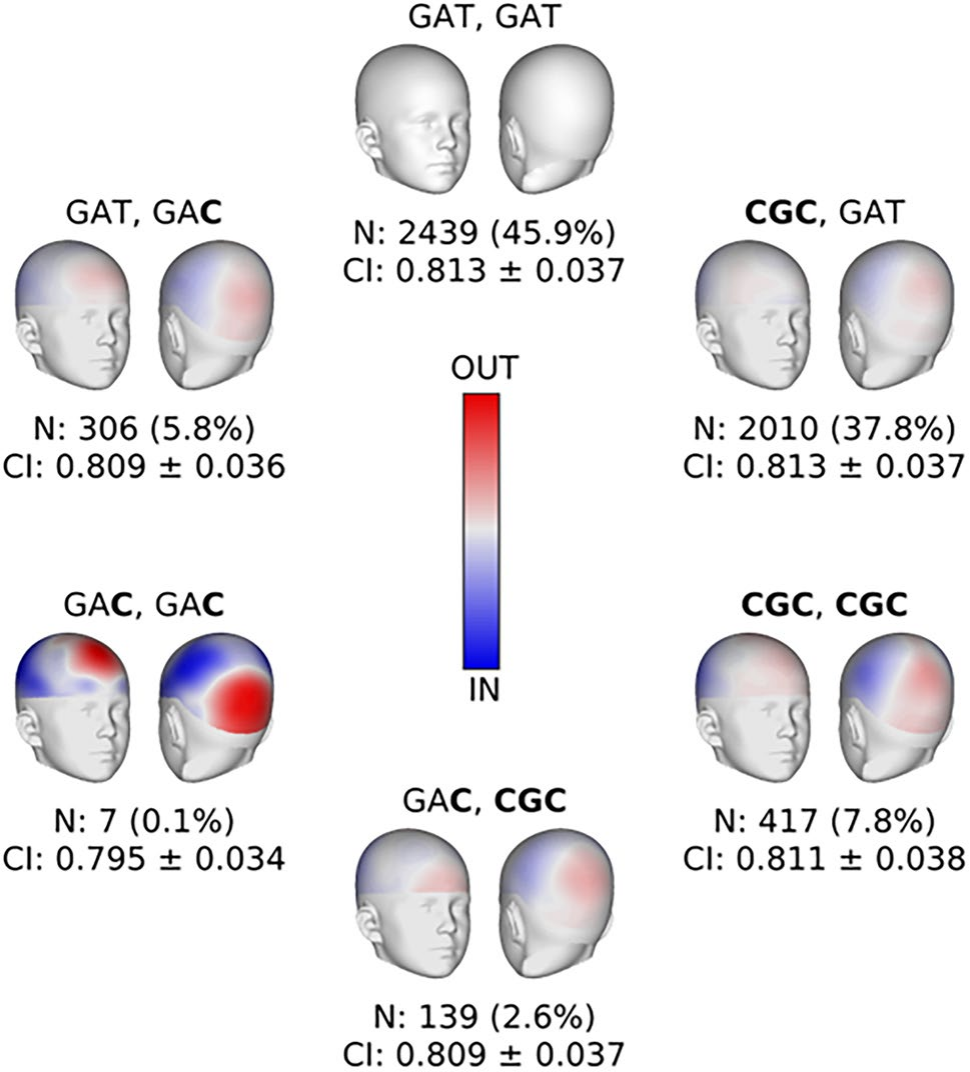
Diplotypes at sagittal nonsyndromic craniosynostosis risk locus near *BMP2* are associated with normal cranial vault shape. Overtransmitted alleles are indicated in bold in the three haplotypes comprising *rs6054763*, *rs932517*, and *rs6054764* respectively*.* For each diploid combination of haplotypes, the average cranial vault shape is compared to the average shape of the homozygous GAT carriers who serve as reference. Red and blue indicate outward protrusion and inward depression respectively, with grey indicating no difference. The colorscale applies to the whole figure. Average and standard deviation of cranial index (CI) are indicated for all diploid groups. N indicates the sample size.

Figure 4 illustrates how the narrowing and elongation of the cranial vault is most pronounced in the homozygous GAC carriers, with the C allele for rs6054764 being the only risk allele on the haplotype. Additionally, cranial index (CI), defined as the ratio of maximal cranial width over maximal cranial length, was significantly lower in carriers of at least one GAC haplotype versus the references (p-value: 0.0252). No significant difference in CI was observed between the reference group and carriers of at least one CGC haplotype, which notably contains all three risk alleles (p-value: 0.3446).

## Discussion

We previously found a signal for sNCS at 20p12.3 in the intergenic space between *BMP2* and *LINC01428* in using a GWAS^10^ and a targeted sequencing study^38^. These findings motivated our current use of WGS data for 63 affected trios, which allowed for additional granularity to identify potential causal variants in this region.

Overall, we identified 24 variants (all with MAF ≥ 0.39) as GWS within the intergenic region; the only variants that were GWS in our entire dataset. Applying an rvTDT analysis did not show the RVs in this region to be significant. This suggests that these common variants themselves are likely, in part, to be causal for the observed locus. It is possible that no single variant is solely causal for the phenotype; instead, the effect may be additive.

Identifying which of these variants were potentially causal within the small haplotype was challenging. rvTDT analysis confirmed that the locus does not appear to be driven by a RV with a large effect, as these would have been captured in set-based tests. Nor does the locus appear to be driven by de novo variants, as no de novo variant in the region was shared by more than two trios. Finally, copy number repeat variation (CNV) analysis failed to identify any associated CNVs and no GWS variants were found to be known eQTLs or pQTLs.

With these findings, our best evidence for approximating causality is around SNVs rs6054763, rs6054764, and rs932517. These variants are predicted to be TFBS and predicted damaging by CADD. Upon running stratified analyses, these variants remained significant after the multiplex families were removed but were not GWS after removing the 23 previously analyzed GWAS trios (although no variant was), which is reasonable after having removed so many trios from a small dataset. However, these variants had the third lowest p-values overall and the lowest along the haplotype, suggesting that the 40 trios not included in our original GWAS are the primary drivers of this locus and provide an internal replication of our GWAS findings for *BMP2*.

The variant rs6054764 is particularly interesting, as it is predicted to be the most deleterious by CADD and is the most highly enriched minor allele, with the minor allele present in 48% of the dataset, compared to 32% in the European population. This variant is also associated with another bone related trait, heel bone mineral density^39^.

In an effort to further identify putative functional noncoding regions, we analyzed ChIP databases. ChIP evidence gives multiple potential TFs as binders, but the most interesting is *CTCF*, which was identified by ChIP in multiple organs/tissue, including bone. *CTCF* is particularly intriguing as our group had previous identified a link between *CTCF* and mNCS^11^. In that study, the most significantly associated SNV, rs6127972 at the *BMP7* locus, was found to reside in a linkage disequilibrium block that overlapped a CTCF TFBS; that binding site was significantly hypomethylated in mesenchymal stem cells derived from fused metopic compared to open sutures^11^. Reduced expression of CTCF itself has been found to cause craniofacial malformations in mice^40^, and further CTCF binding sites have been localized near other genes that direct craniofacial development^41^. Other TFs identified by ChIP at this 800 bp region also have interesting biological evidence. FOS, for which both rs6054764 and rs6054763 are TFBS is known to be increased in the *FGF2* pathway that stimulates premature cranial suture fusion^42^. RAD51 is a known risk gene for orofacial clefts^43^, and mutations in EP300 are responsible for Rubinstein–Taybi syndrome, which causes craniofacial defects^44^.

It is also possible that this region is modulating the expression of either *BMP2* or *LINC01428*. There has been little published regarding the function of the lncRNA gene. lncRNAs have been found to play a role in NCS; the lncRNA HOTAIR, which has previous associations with osteogenesis and osteoarthritis, was recently found to be dysregulated in NCS and results in impaired osteoclast differentiation^45^. Further, the master bone transcription factor *RUNX2* is modulated by lncRNAs, though not the particular lncRNA associated in our study^46^. Conversely, it is likely that our three variants of interest are not solely responsible for the locus; with the most probable explanation being some sort of additive effect caused by multiple potential causal variants in the region.

An interesting item of note is that we did not see a significant variant at *BBS9* on chromosome 7, which had been previously observed in our original GWAS^10,38^. The association is still present, but at a less significant p-value (lowest p-value = 9e − 6) than we previously observed, although effect size remained consistent. This may be due to our WGS dataset being much smaller than our GWAS and targeted sequencing datasets in which we previously identified the *BBS9* association. Additionally, the WGS trios were selected for sequencing based on the chromosome 20 (*BMP2*) association, which was always stronger, and not the chromosome 7 (*BBS9*) association. To combat potential bias of the previously analyzed families, we performed the additional stratified analysis removing these families.

We further found that our three putative associated variants near *BMP2* (alone and in combination) also impacted normal-range human cranial vault shape. Specifically, we found that these variants were associated with a more dolichocephalic shape, echoing the more severe phenotype observed in sNCS and validating our initial hypothesis. These results suggest that normal-range and dysmorphic variation are genetically linked and may be best conceptualized as a continuum. Because our putative variants are relatively common, we expect them to be present in a sizable portion of unaffected individuals. The dolichocephalic tendency we observed suggests an effect on the sagittal suture. A key factor may be timing. Due to rapid brain growth early in life, pre- or peri-natal suture fusion leads to the kinds of severe phenotypic outcomes we typically associate with craniosynostosis. If this fusion is delayed, however, the child may show more subtle changes in vault shape, with few or no other sequalae, and likely within the normal range of variation. This could be considered a subclinical manifestation of craniosynostosis. Two independent lines of evidence support this claim. First, a recent report found that previously undocumented fusion of the sagittal suture is present on CT scans in about 5% of children, none of whom exhibited clinical symptoms of sNSC^47^. Second, this type of “delayed-onset” craniosynostosis has been reported in a congenital rabbit model and shows an intermediate phenotype between early onset and unaffected animals^48^. Similar subclinical manifestations have been reported in orofacial clefting^49^. Thus, this kind of phenotypic variability should be expected in complex and multifactorial traits, where the genotype-to-phenotype correlation is heavily modulated.

In summary, we identified a highly significant association in the intergenic region between *BMP2* and *LINC01428*. Identification of potential causal variants proved challenging, as the region was noncoding, no RV association was detected, and none of the significant variants were QTLs. By using CADD scores and TFBS probabilities, we propose that the most likely causal variants are rs6054763, rs6054764, and rs932517, which are predicted deleterious, highly enriched in probands, and probably TFBS. Because of the challenges in attributing potential causality to noncoding variants such as these, future work on these families and larger, more diverse population samples could focus on other genomic analysis, such as copy number variation and other types of omics data, particularly RNA-seq or ATAC-seq analysis, which would allow for accurate quantification of RNA expression levels and chromatin accessibility regions.

### Supplementary Information

Supplementary Figures.

Supplementary Tables.

## Data Availability

The data analyzed and reported in this manuscript is publicly available and can be accessed from the database of Genotypes and Phenotypes (dbGaP) and from the Kids First Data Resource Center (https://kidsfirstdrc.org). Data used in the preparation of this article were obtained from the Adolescent Brain Cognitive Development (ABCD) Study (https://abcdstudy.org), held in the NIMH Data Archive (NDA). This is a multisite, longitudinal study designed to recruit more than 10,000 children age 9–10 and follow them over 10 years into early adulthood. The ABCD Study is supported by the National Institutes of Health and additional federal partners under award numbers U01DA041048, U01DA050989, U01DA051016, U01DA041022, U01DA051018, U01DA051037, U01DA050987, U01DA041174, U01DA041106, U01DA041117, U01DA041028, U01DA041134, U01DA050988, U01DA051039, U01DA041156, U01DA041025, U01DA041120, U01DA051038, U01DA041148, U01DA041093, U01DA041089, U24DA041123, U24DA041147. A full list of supporters is available at https://abcdstudy.org/federal-partners.html. A listing of participating sites and a complete listing of the study investigators can be found at https://abcdstudy.org/consortium_members/. ABCD consortium investigators designed and implemented the study and/or provided data but did not necessarily participate in analysis or writing of this report. This manuscript reflects the views of the authors and may not reflect the opinions or views of the NIH or ABCD consortium investigators. The ABCD data repository grows and changes over time. The ABCD data used in this report came from 10.15154/1519007. DOIs can be found at 10.15154/1519007. The resources and services used in this work were provided by the VSC (Flemish Supercomputer Center), funded by the Research Foundation-Flanders (FWO) and the Flemish Government.
