# Supplementary material for: Whole genome sequencing identifies associations for nonsyndromic sagittal craniosynostosis with the intergenic region of BMP2 and noncoding RNA gene LINC01428
Source: Sci Rep. 2024 Apr 12;14:8533. doi: 10.1038/s41598-024-58343-w (PMC11014861; doi:10.1038/s41598-024-58343-w)
Supplement: Supplementary file 1 — Supplementary Figures. [file 41598_2024_58343_MOESM1_ESM.docx]

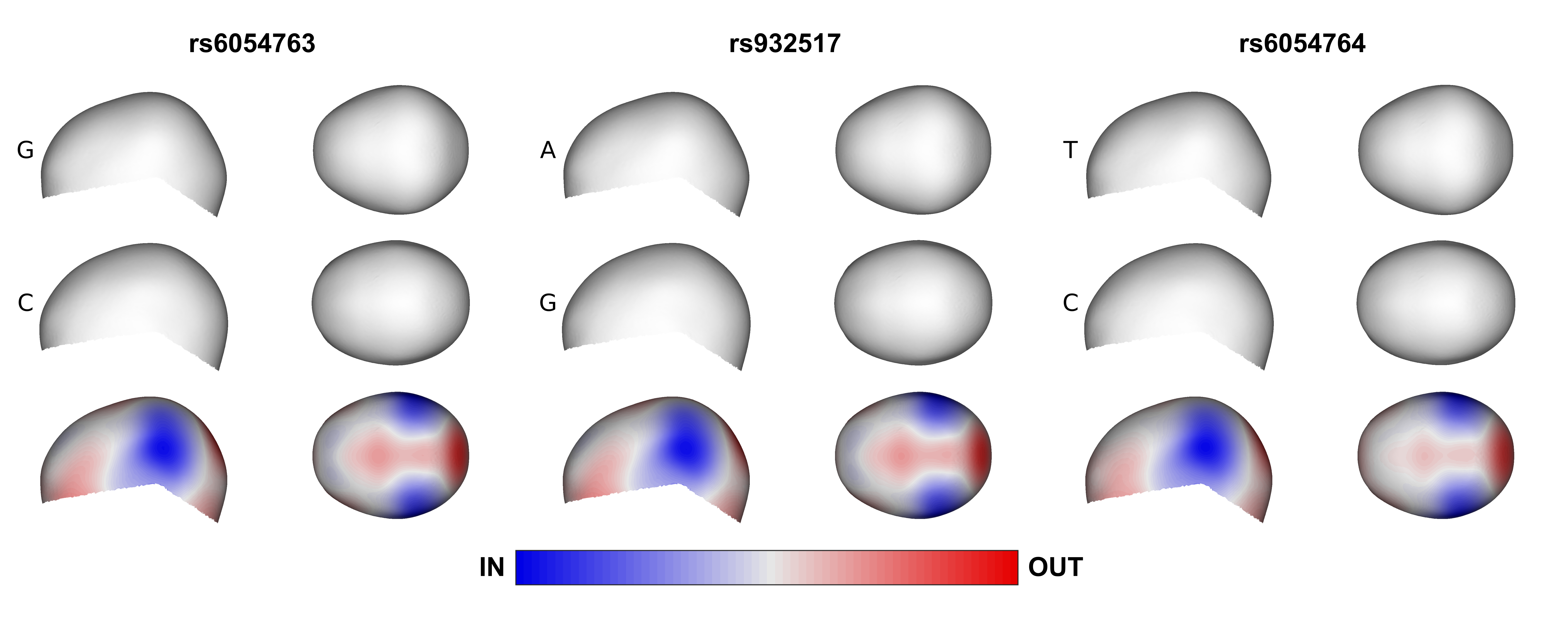


**Supplemental Figure 1 – Sagittal Nonsyndromic Craniosynostosis Risk Single Nucleotide Variants Associated with Cranial Vault Shape Variation in the General Population.** The cranial vault surface shows the effect of rs6054763, rs932517, and rs6054764 respectively, exaggerated in the direction of the major (top row) and minor (middle row) allele. Red and blue indicate outward protrusion and inward depression respectively, with grey indicating no difference.
